# Multi-kingdom characterization of the core equine fecal microbiota based on multiple equine (sub)species

**DOI:** 10.1186/s42523-020-0023-1

**Published:** 2020-02-12

**Authors:** J. E. Edwards, S. A. Shetty, P. van den Berg, F. Burden, D. A. van Doorn, W. F. Pellikaan, J. Dijkstra, H. Smidt

**Affiliations:** 1 Laboratory of Microbiology, Wageningen University & Research, Wageningen, 6708 WE Netherlands; 2 The Donkey Sanctuary, Sidmouth, Devon EX10 ONU UK; 3 Division of Nutrition, Department of Farm Animal Health, Faculty of Veterinary Medicine, Utrecht University, Utrecht, 3584 CM Netherlands; 4 Department of Equine Health, Faculty of Veterinary Medicine, Utrecht University, Utrecht, 3584 CL Netherlands; 5 Animal Nutrition Group, Wageningen University & Research, Wageningen, 6708 WD Netherlands

**Keywords:** Feces, Barcoded amplicon sequencing, Bacteria, Archaea, Anaerobic fungi, Horse, Donkey, Mule, Hinny, Zebra

## Abstract

**Background:**

Equine gut microbiology studies to date have primarily focused on horses and ponies, which represent only one of the eight extant equine species. This is despite asses and mules comprising almost half of the world’s domesticated equines, and donkeys being superior to horses/ponies in their ability to degrade dietary fiber. Limited attention has also been given to commensal anaerobic fungi and archaea even though anaerobic fungi are potent fiber degrading organisms, the activity of which is enhanced by methanogenic archaea. Therefore, the objective of this study was to broaden the current knowledge of bacterial, anaerobic fungal and archaeal diversity of the equine fecal microbiota to multiple species of equines. Core taxa shared by all the equine fecal samples (*n* = 70) were determined and an overview given of the microbiota across different equine types (horse, donkey, horse × donkey and zebra).

**Results:**

Equine type was associated with differences in both fecal microbial concentrations and community composition. Donkey was generally most distinct from the other equine types, with horse and zebra not differing. Despite this, a common bacterial core of eight OTUs (out of 2070) and 16 genus level groupings (out of 231) was found in all the fecal samples. This bacterial core represented a much larger proportion of the equine fecal microbiota than previously reported, primarily due to the detection of predominant core taxa belonging to the phyla *Kiritimatiellaeota* (formerly *Verrucomicrobia* subdivision 5) and *Spirochaetes*. The majority of the core bacterial taxa lack cultured representation. Archaea and anaerobic fungi were present in all animals, however, no core taxon was detected for either despite several taxa being prevalent and predominant.

**Conclusions:**

Whilst differences were observed between equine types, a core fecal microbiota existed across all the equines. This core was composed primarily of a few predominant bacterial taxa, the majority of which are novel and lack cultured representation. The lack of microbial cultures representing the predominant taxa needs to be addressed, as their availability is essential to gain fundamental knowledge of the microbial functions that underpin the equine hindgut ecosystem.

## Background

The hindgut microbiome is key to the ability of equines to degrade dietary fiber, as equines themselves lack fiber degrading enzymes. In recent years, there has been a move to characterizing the equine hindgut microbiota using high throughput sequencing of fecal [1–7] and digesta samples [8, 9], as well as determining the equine fecal and hindgut core composition [3, 7, 8, 10, 11]. This is particularly important considering the variation reported in the hindgut microbiota and metabolome of different animals [12]. By determining the core microbiota of healthy equines, insights can be gained into cornerstone taxa and functions present in the ecosystem [13, 14].

Whilst the equine hindgut bacterial core has been described, its composition differs between studies [7, 8, 10, 11]. To date, it has been concluded that diet changes the composition and size of the equine bacterial core [10], unlike age and obesity which have minimal effects [7, 10]. The bacterial core has also been shown to be represented by multiple low abundance taxa, which together are of small cumulative abundance [7, 8, 10]. However, studies of the bacterial core to date have focused only on domesticated horses and/or ponies, which represent only one of the eight extant species of the equid family [15].

The knowledge of the gut microbiota from horses and ponies is normally directly translated to donkeys and mules, which comprise 47.9% of the world’s 113 million domesticated equines [16]. The validity of this direct translation is not clear particularly as diet and gut transit time, which are key influencing factors of the hindgut microbiome, differ between horses/ponies and donkeys [17, 18]. Differences also occur in feed digestibility, with donkeys relative to ponies consistently having a higher dry matter digestibility for a given diet [18].

Furthermore, bacteria are not alone in the equine hindgut, as anaerobic fungi, methanogenic archaea and protozoa are also present [19]. Bacteria as well as anaerobic fungi are the primary degraders of fiber in mammalian herbivores. However, anaerobic fungi are significantly better at degrading plant cell walls than bacteria [20], due to their invasive growth and potent fiber degrading enzymes [21, 22]. Methanogenic archaea do not degrade fiber, but are known to promote anaerobic fungal activity due to the removal of fermentation end products [23]. Protozoa have been suggested not to play a major role in hindgut fiber degradation, as cellulose digestion is not changed by their removal [24].

The majority of knowledge on anaerobic fungi and methanogenic archaea in the gut of mammalian herbivores is based on ruminants, with only limited information on equines [19]. There is evidence that equine anaerobic fungi are distinct from those found in ruminants, both in terms of taxonomy and physiology [25, 26]. In terms of methanogenic archaea, sequencing based studies of fecal samples have indicated that the genera *Methanocorpusculum* and *Methanobrevibacter* predominate [6, 27]. It remains to be verified, however, if these two genera are both part of the core hindgut microbiota of equines.

Protozoa present in the equine hindgut are similar to those present in the rumen of ruminants in terms of being dominated by ciliates, although amoeboid and flagellated protozoa may also be present [19]. The majority of the 24 ciliate genera that have been described in equines to date, however, are not typically found in ruminants. The two ciliate protozoal genera *Blepharocorys* and *Cycloposthium* are thought to be the most prevalent in equines [19]. In recent years, two studies have used molecular methods based on rumen protozoal derived 18S rRNA gene primers to look at protozoal concentrations and diversity in the equine hindgut [6, 28]. However, caution is needed as the suitability of these rumen protozoal derived primers for study of equine ciliate protozoa remains to be verified. This can only be done once 18S rRNA gene sequences for the numerous equine protozoal genera described to date are available. As such, analysis of equine protozoa was not performed as part of this multi-kingdom study.

The objective of this study was, therefore, to determine the bacterial, archaeal and anaerobic fungal composition of fecal samples from a large cohort of equines (*n* = 70) that included five extant species (i.e. *Equus ferus*, *Equus africanus*, *Equus quagga*, *Equus zebra* and *Equus greyvii*), as well as mules and hinnies (i.e. horse × donkey). All animals were fed pasture or hay/haylage based diets and, in some cases, received complementary feedstuffs (< 1 kg/day) in order to meet dietary requirements (Additional file 1: Table S1). In order to capture as much variation as possible, the animals were sourced from multiple geographical locations. From this dataset an overview of the microbiota across equine types is given, followed by identification of the core bacteria, anaerobic fungi and archaea.

## Results

The equine cohort (*n* = 70) studied here was composed of healthy animals aged between 4 and 26 years that had no known history of any gut mediated disease. The cohort included horses and ponies (*Equus ferus caballus*, *n* = 18), donkeys (*Equus africanus asinus*, n = 18), hybrids of donkey and horse (*Equus ferus caballus* × *Equus africanus asinus*, n = 18) and several different (sub)species of zebras (*Equus quagga burchellii*, *n* = 2; *Equus quagga boehmi*, *n* = 8; *Equus zebra hartmannae n* = 3; *Equus greyvii*, n = 3). Details of the individual animals including their location, diet and management are given in Additional file 1: Table S1. For the purpose of providing an overview of the fecal microbiota across equine types, the animals were classed as being either horse (n = 18), donkey (n = 18), horse × donkey (n = 18) or zebra (*n* = 16).

### Microbial concentrations

**Fig. 1:**
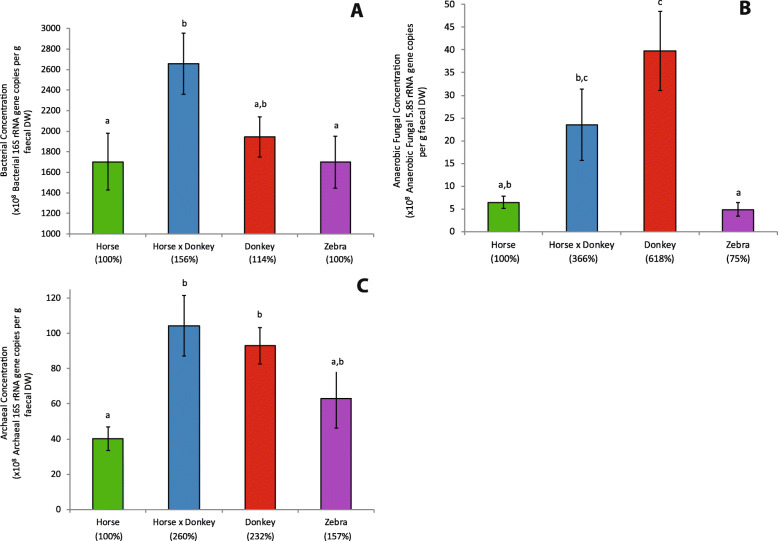
Effect of equine type on the fecal bacterial (**a**), anaerobic fungal (**b**) and archaeal (**c**) concentrations on a dry weight basis. Columns represent the mean (*n* = 18, except for zebra where *n* = 16) and error bars the SEM. Letters above the bars within each plot indicate significant differences (*P* < 0.05). Percentages stated on the x-axis in brackets indicate how the mean of each equine type compared to that of the horse.

Due to equine type being associated with differences in fecal dry matter content (*P* = 0.006; Additional file 2: Figure S1), where zebra fecal dry matter was significantly higher than that of horse and of donkey, microbial concentration data were analyzed on a dry weight basis. Equine type was associated with differences in fecal bacterial concentrations (*P* = 0.016), with horse and zebra both being significantly lower than horse × donkey, and donkey being intermediate (Fig. 1a). Equine type was also associated with fecal anaerobic fungal concentrations (*P* < 0.001), with donkey having over 6-fold and 8-fold higher concentrations than horse and zebra, respectively (Fig. 1b). Horse × donkey had an almost 5-fold higher anaerobic fungal concentration than zebra. Fecal archaeal concentrations were also associated with equine type (*P* < 0.001), with donkey and horse × donkey having significantly higher archaeal concentrations than horse, and zebra not significantly differing from any of the other equine types (Fig. 1c). Analysis of the same data on a fresh weight basis showed generally similar trends (Additional file 3: Figure S2).

### Prokaryotic community composition

The bacteria (mean ± standard deviation (SD): 96.2 ± 3.08% of the 16S rRNA gene sequences) were represented by 2070 different OTUs (operational taxonomic units) which could be summarized to 231 different genus-level phylogenetic groupings. The archaea (3.8 ± 3.08% of the 16S rRNA sequences) were represented by 48 different OTUs, which could be summarized into seven different genus-level phylogenetic groupings. Of the 17 phyla detected, the following six were the most predominant: *Firmicutes*, *Bacteroidetes*, *Verrucomicrobia*, *Spirochaetes*, *Fibrobacteres* and *Euryarchaeota* (Additional file 4: Figure S3). Of the 76 families that could be classified, the most predominant families included the *Lachnospiraceae*, *Ruminococcaceae*, *Bacteroidales* S24 − 7_group, *Spirochaetaceae* and *Fibrobacteraceae* (Additional file 5: Figure S4).

Differences in fecal prokaryotic alpha diversity was associated with equine type in terms of both the number of observed OTUs (*P* = 0.007) and Phylogenetic Diversity (*P* = 0.029). Donkey fecal microbiota had a lower number of observed OTUs (228 ± 28 OTUs) compared to that of horse (253 ± 28 OTUs), horse × donkey (258 ± 28 OTUs) and zebra (255 ± 26 OTUs). Donkey fecal microbiota also had a lower Phylogenetic Diversity (18.96 ± 1.142) compared to that of zebras (20.19 ± 1.032), with neither donkey nor zebra significantly differing from horse (19.34 ± 1.396) or horse × donkey (19.43 ± 1.097).

**Fig. 2:**
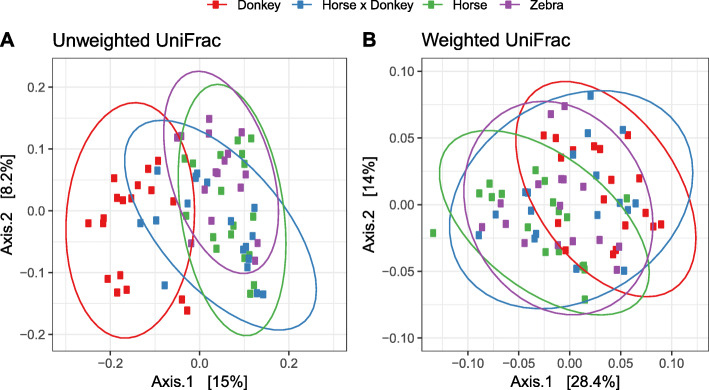
Unweighted (**a**) and weighted (**b**) UniFrac based principal co-ordinates analysis of the fecal prokaryotic community composition of the different equine types at the OTU level. Analysis used Log_10_ transformed data with ellipses representing 95% confidence intervals, and the percentages values labelled on each axis indicating the amount of total variation represented.

Beta diversity analysis of the fecal microbiota was performed using a non-constrained principal co-ordinate analysis (PCoA) at the OTU level based on pairwise UniFrac distances. Donkey fecal microbiota separated from the other equine types in the unweighted analysis but not the weighted analysis (Fig. 2). This suggests that the largest variation in the dataset was associated with donkey specific taxa occurring at a low relative abundance.

**Fig. 3:**
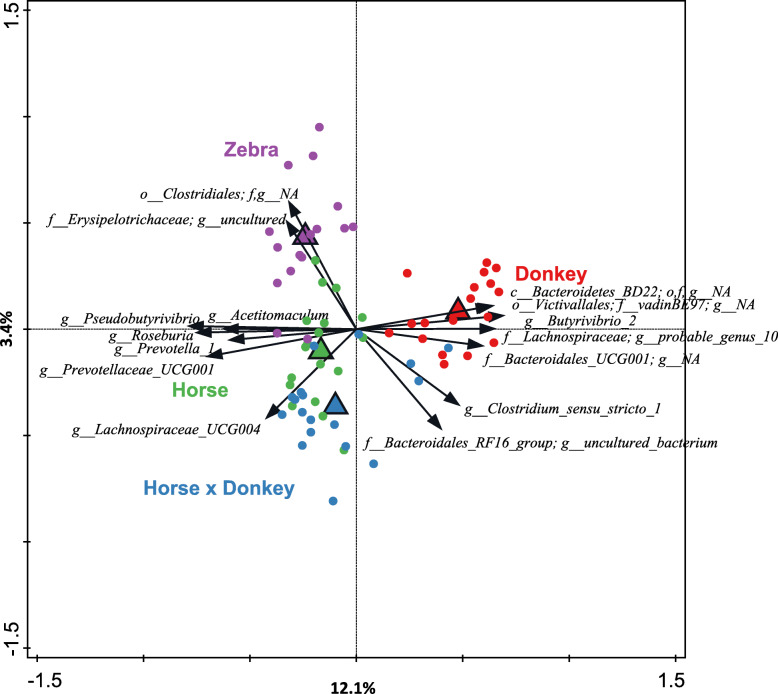
Redundancy analysis triplot showing the relationship between the top fifteen prokaryotic genus-level phylogenetic groupings of the OTUs for which the variation is best explained by the constrained axes. Arrow length indicates the variance that can be explained by equine type, with the perpendicular distance of the equine types to the arrow indicating the relative abundance of the genus-level phylogenetic grouping. Arrow labels indicate the taxonomic affiliation of genus-level phylogenetic groups, with the level (i.e. class (c), order (o), family (f) or genus (g)) and taxon (as defined by the Silva 16S rRNA database) that the groups could be reliably assigned to. For example ‘*g_Prevotella_1*’ represents an OTU reliably assigned to the *Prevotella_1* genus, whereas “*c_Bacteroidetes_BD2–2; o,f,g_NA*” was reliably assigned to the class *Bacteroidetes_BD2–2* but the order, family and genus could not be annotated (NA). Triangular symbols indicate the equine type means and circle symbols the individual samples color coded by equine type. Equine type explained 18.3% of the total variation in the dataset, and the plot axis are labelled with the amount of this they represent.

Redundancy analysis using genus-level phylogenetic groups confirmed that equine type was associated with differences in the prokaryotic community composition (*P* = 0.002), with equine type accounting for 18.3% of the total variation in the dataset (Fig. 3). The majority of the variation was represented by the first canonical axis, which showed that the prokaryotic community composition of donkey fecal microbiota separated from that of the three other equine types, the latter separating along the second canonical axis.

Five genus-level phylogenetic groups appeared to be positively associated with donkey. Of these five, two could be annotated to the genus level (*Butyrivibrio* 2, *Lachnospiraceae* probable_genus_10) and the others only to family (*Bacteroidales* UCG-001; *Victivallales* family_vadinBE97) or class level (*Bacteroidetes* BD2–2). Several genera appeared to generally be positively associated with horse, zebra and horse × donkey (and conversely negatively associated with donkey). These included *Pseudobutyrivibrio*, *Roseburia*, *Prevotella* 1, *Acetitomaculum* and *Prevotellaceae* UCG-001. The genus *Lachnospiraceae* UCG-004 appeared to be positively associated with horse × donkey and horse to different extents. An uncultured genus from the family *Erysipelotrichaceae* and an unknown family within the order *Clostridiales* appeared to be positively associated with zebra.

### Anaerobic fungal community composition

For the analysis of anaerobic fungal community composition, reproducible PCR products of sufficient amount for sequencing were only obtained from 64 of the 70 different animals. The three horse and three zebra samples that failed (H4, H6, H7, Z11, Z14 & Z16: Additional file 1: Table S1) had the lowest anaerobic fungal concentrations detected in the cohort. Therefore, these six samples were considered to be below the detection limit of the amplicon sequencing method used in our study. From the 64 samples there were 358 OTUs detected, and these could be summarized to eight different genera. The taxonomic framework used in the anaerobic fungal ITS1 (AF-ITS1) database also includes as yet uncharacterized genus- or species-level clades [29]. Whilst the 358 OTUs could be summarized to nine clades, no further analysis at the clade level was performed as 53% of the OTUs could not be annotated at this level (Additional file 6: Figure S5). Of the eight genera detected that could be annotated (AL1, AL7, *Caecomyces*, KF1, *Neocallimastix*, *Piromyces*, SK1 and SK3), *Caecomyces* and the uncultivated genus AL1 predominated the dataset overall (Additional file 7: Figure S6). The other six genera were predominant and/or present in only a few individual animals (Additional file 7: Figure S6).

Differences in fecal anaerobic fungal alpha diversity was associated with equine type in terms of the number observed OTUs (*P* = 0.006), but not Phylogenetic Diversity (*P* = 0.989). Horse had a higher number of observed OTUs (22 ± 5 OTUs) compared to both donkey (16 ± 5 OTUs) and horse × donkey (16 ± 6 OTUs), with zebra (20 ± 6 OTUs) not differing from any of the other equine types.

**Fig. 4:**
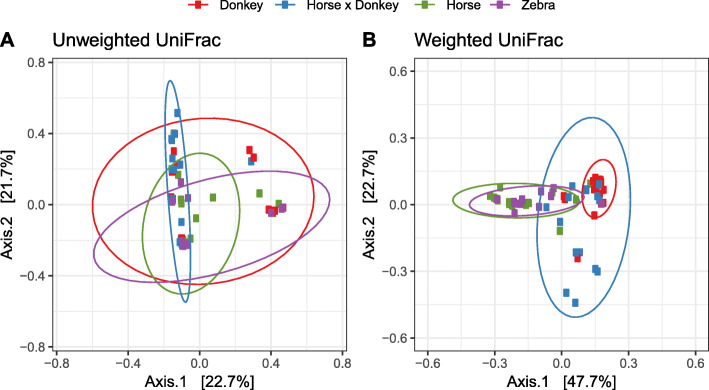
Unweighted (**a**) and weighted (**b**) UniFrac based principal co-ordinates analysis of the fecal anaerobic fungal community composition of the different equine types at the OTU level. Analysis used Log_10_ transformed data with ellipses representing 95% confidence intervals, and the percentages values labelled on each axis indicating the amount of total variation represented.

Beta diversity analysis of the fecal anaerobic fungal community using PCoA showed that all the equine types overlapped to some extent in the unweighted analysis (Fig. 4a). However, in the weighted PCoA the horse and zebra sample groups clustered separately from the donkey group and most variation was seen in the horse × donkey sample group (Fig. 4b).

**Fig. 5:**
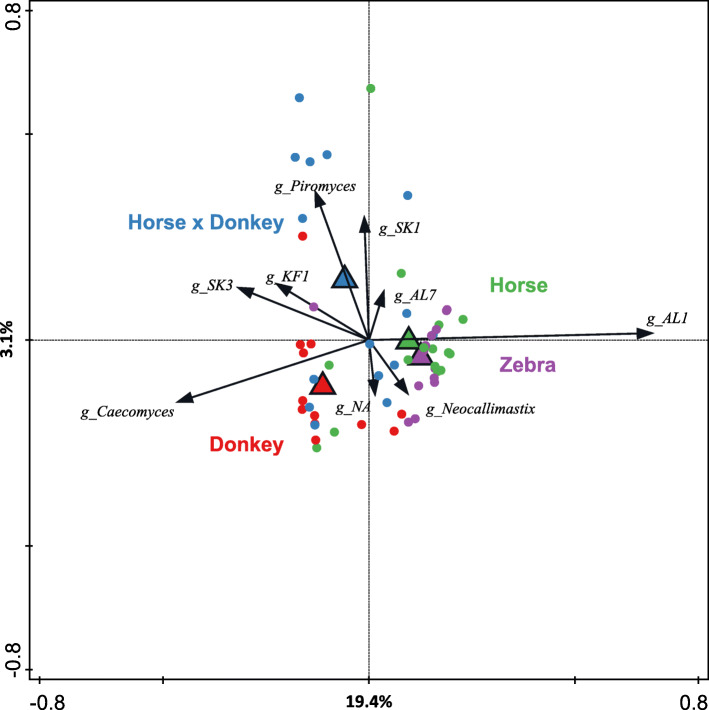
Redundancy analysis triplot showing the relationship between the anaerobic fungal genus-level phylogenetic groupings of the OTUs for which the variation is best explained by the constrained axes. Arrow length indicates the variance that can be explained by equine type, with the perpendicular distance of the equine types to the arrow indicating the relative abundance of the genus-level phylogenetic grouping. Arrow labels indicate the taxonomic affiliation that the genera could be reliably assigned to. For example ‘g_AL1’ represents a grouping reliably assigned to the AL1 genus, whereas ‘g_NA’ indicates that it was reliably assigned to the family *Neocallimastigaceae* but the genus could not be annotated (NA). Triangular symbols indicate the equine type means and circle symbols the individual samples color coded by equine type. Equine type explained 23.6% of the total variation in the dataset, and the plot axis are labelled with the amount of this they represent.

Redundancy analysis using genus-level phylogenetic groups confirmed that equine type was associated with differences in the anaerobic fungal community composition (*P* = 0.002) and accounted for 23.6% of the total variation in the dataset (Fig. 5). Horse and zebra were positively associated with the genus AL1, whereas donkey was most positively associated with *Caecomyces* and the horse × donkey with *Piromyces* and SK1.

### Equine Core microbiota analysis

**Fig. 6:**
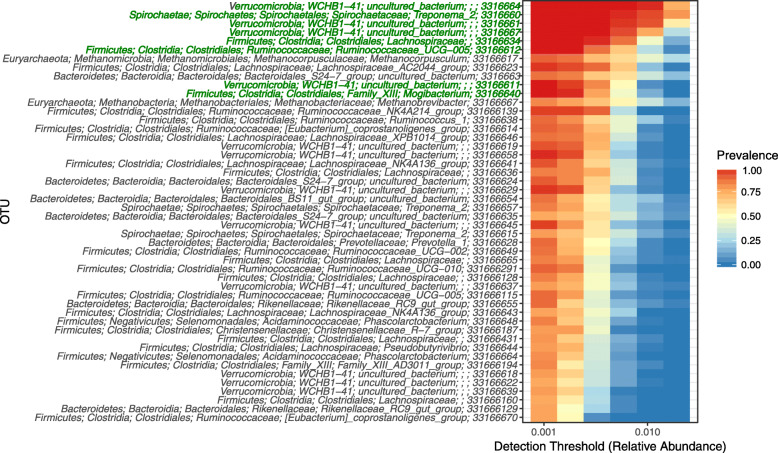
Heat map showing the relative abundance (> 0.001 cut-off) and prevalence (75% cut-off) of the prokaryotic OTUs in the 70 equine fecal samples analyzed. Different detection thresholds are used, providing information regarding the relative abundance of the OTUs relative to their prevalence. Taxonomic assignments of the OTUs are given to five taxonomic ranks (phylum, class, order, family and genus) where possible, followed by the OTU ID number. Where this was not possible, the non-annotated ranks were left empty (e.g. *Verrucomicrobia*; WCHB1–41; uncultured_bacterium;;; 3316664 has no information for the family and genus ranks). OTUs present in all animals (i.e. core) have their taxonomic assignments written in green.

Analysis of the prokaryotic community composition at the OTU level indicated that of the 2118 OTUs detected, only 48 OTUs were present in at least 75% of the animals when a cut-off of > 0.001 was used (Fig. 6). Of these 48 OTUs, only eight OTUs were core, i.e. found in every animal (Fig. 6). Four of these OTUs belonged to an uncharacterized class, WCHB1–41, in the *Verrucomicrobia* phylum. The other four had annotation at lower taxonomic ranks: a non-annotated genus within the *Lachnospiraceae*, an uncharacterized genus called *Ruminococcaceae* UCG-005 and the two characterized genera *Mogibacterium* and *Treponema* 2. WCHB1–41 and *Treponema* 2 were the most abundant of the eight core OTUs (Table 1). These eight core OTUs represented 13.4% ± 3.26 (mean ± SD) of the prokaryotic community.

**Table 1:** Taxonomic annotation^a^ and percentage relative abundances^b^ of the core taxa detected at the OTU level

Phylum	Class	Order	Family	Genus	OTU No.	Mean	SD	SEM
*Spirochaetae*	*Spirochaetes*	*Spirochaetales*	*Spirochaetaceae*	*Treponema* 2	3316660	3.41	1.74	0.21
*Verrucomicrobia*	WCHB1–41	uncultured bacterium	NA	NA	3316664	2.76	1.26	0.15
*Verrucomicrobia*	WCHB1–41	uncultured bacterium	NA	NA	3316661	2.26	1.16	0.14
*Verrucomicrobia*	WCHB1–41	uncultured bacterium	NA	NA	3316667	1.56	0.71	0.08
*Firmicutes*	*Clostridia*	*Clostridiales*	*Lachnospiraceae*	NA	33166634	1.26	0.84	0.10
*Firmicutes*	*Clostridia*	*Clostridiales*	*Ruminococcaceae*	*Ruminococcaceae* UCG − 005	33166612	0.88	0.62	0.07
*Verrucomicrobia*	WCHB1–41	uncultured bacterium	NA	NA	33166611	0.62	0.30	0.04
*Firmicutes*	*Clostridia*	*Clostridiales*	Family XIII	*Mogibacterium*	33166640	0.61	0.32	0.04

^a^NA indicates that the taxonomic rank could not be annotated

^b^Percentage relative abundances are given in terms of the mean (*n* = 70), standard deviation (SD) and standard error of the mean (SEM)

**Fig. 7:**
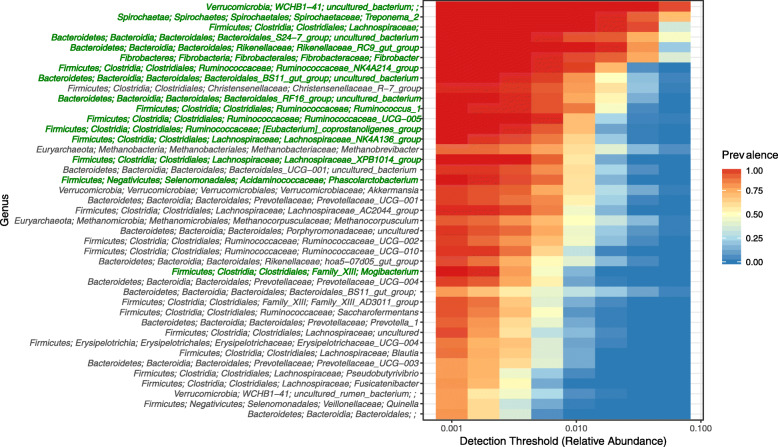
Heat map showing the relative abundance (> 0.001 cut-off) and prevalence (75% cut-off) of the prokaryotic genus level OTU groups in the 70 equine fecal samples analyzed. Different detection thresholds are used, providing information regarding the relative abundance of the genus level OTU groups relative to their prevalence. Taxonomic assignments of the genera are given to five taxonomic ranks (phylum, class, order, family and genus) where possible. Where this was not possible, the non-annotated ranks were left empty (e.g. *Verrucomicrobia*; WCHB1–41; uncultured_bacterium;;; has no information for the family and genus ranks). Genus level groups present in all animals have their taxonomic assignments written in green.

Similar analysis performed with the OTUs grouped at the genus level showed that 41 genus level groups were present in at least 75% of the animals when a cut-off of > 0.001 was used (Fig. 7). Of these 41 genus level groups, only 16 were core (Fig. 7**;** Table 2). Of the core genus level groups only five could be annotated to characterized genera: *Treponema* 2, *Fibrobacter*, *Ruminococcus* 1, *Phascolarctobacterium* and *Mogibacterium*. In contrast, little is known about the other 11 genus level groups which were represented by uncharacterized taxa at the genus (nine groups), family (one group) and class (one group) level. The two most predominant core genus level groups, consistent with the OTU based analysis, were an uncharacterized class belonging to the uncultivated class WCHB1–41 within the *Verrucomicrobia* and *Treponema* 2 (Table 2). These 16 core genus level groups represented 61.2% ± 8.35 (mean ± SD) of the prokaryotic community.

**Table 2:** Taxonomic annotation^a^ and percentage relative abundances^b^ of the core taxa detected at the genus level

Phylum	Class	Order	Family	Genus	Mean	SD	SEM
*Verrucomicrobia*	WCHB1–41	uncultured bacterium	NA	NA	12.79	4.36	0.52
*Spirochaetae*	*Spirochaetes*	*Spirochaetales*	*Spirochaetaceae*	*Treponema* 2	8.35	3.81	0.46
*Bacteroidetes*	*Bacteroidia*	*Bacteroidales*	*Bacteroidales* S24–7 group	uncultured bacterium	6.93	4.51	0.54
*Fibrobacteres*	*Fibrobacteria*	*Fibrobacterales*	*Fibrobacteraceae*	*Fibrobacter*	6.33	4.13	0.49
*Firmicutes*	*Clostridia*	*Clostridiales*	*Lachnospiraceae*	NA	5.73	1.97	0.23
*Bacteroidetes*	*Bacteroidia*	*Bacteroidales*	*Rikenellaceae*	*Rikenellaceae* RC9 gut group	4.96	1.88	0.22
*Firmicutes*	*Clostridia*	*Clostridiales*	*Ruminococcaceae*	*Ruminococcaceae* NK4A214 group	2.35	0.94	0.11
*Bacteroidetes*	*Bacteroidia*	*Bacteroidales*	*Bacteroidales* BS11 gut group	uncultured bacterium	2.32	1.57	0.19
*Bacteroidetes*	*Bacteroidia*	*Bacteroidales*	*Bacteroidales* RF16 group	uncultured bacterium	2.01	1.14	0.14
*Firmicutes*	*Clostridia*	*Clostridiales*	*Ruminococcaceae*	*Ruminococcus* 1	1.88	0.86	0.10
*Firmicutes*	*Clostridia*	*Clostridiales*	*Ruminococcaceae*	*Ruminococcaceae* UCG-005	1.58	0.94	0.11
*Firmicutes*	*Clostridia*	*Clostridiales*	*Ruminococcaceae*	*[Eubacterium] coprostanoligenes* group	1.36	0.60	0.07
*Firmicutes*	*Clostridia*	*Clostridiales*	*Lachnospiraceae*	*Lachnospiraceae* NK4A136 group	1.36	0.73	0.09
*Firmicutes*	*Clostridia*	*Clostridiales*	*Lachnospiraceae*	*Lachnospiraceae* XPB1014 group	1.29	0.66	0.08
*Firmicutes*	*Negativicutes*	*Selenomonadales*	*Acidaminococcaceae*	*Phascolarctobacterium*	1.29	0.66	0.08
*Firmicutes*	*Clostridia*	*Clostridiales*	Family XIII	*Mogibacterium*	0.65	0.36	0.04

^a^NA indicates that the taxonomic rank could not be annotated

^b^Percentage relative abundances are given in terms of the mean (*n* = 70), standard deviation (SD) and standard error of the mean (SEM)

No methanogenic archaeal taxon was present in all equines, neither at OTU- nor genus level. Of the seven archaeal genera, *Methanocorpusculum* (91.4%) and *Methanobrevibacter* (88.6%) were most prevalent in the animals sampled (Fig. 7), and represented (mean ± SD) 44.7 ± 30.83% and 51.2 ± 30.41% of the archaeal 16S rRNA gene sequences detected per animal, respectively. The large variation in the mean relative abundance was mainly due to the predominance of one of these two genera compared to the other, or in some animals only *Methanocorpusculum* (6 animals) or *Methanobrevibacter* (5 animals) being present. The other archaeal genera were all lower in prevalence (< 22.9%), and all represented a much smaller proportion (< 0.98%) of the archaeal 16S rRNA gene sequences detected per animal.

Similarly, no core anaerobic fungal OTUs or genera were observed across all equines. Of the eight genera *Caecomyces* (95.3%) and ALI (53.1%) were most predominant in terms of prevalence in the animals sampled, and represented (mean ± SD) 48.2 ± 39.26% and 31.6 ± 39.11% of the anaerobic fungal sequences detected per animal, respectively. The large variation in the mean relative abundance was due to animals being often predominated by only one of these two anaerobic fungal genera (Additional File 7: Figure S6). The other anaerobic fungal genera were all lower in prevalence (< 18.8%) and overall represented a much smaller proportion (< 5.62%) of the mean anaerobic fungal sequences detected per animal.

## Discussion

The objectives of this study were (i) to give an overview of the fecal microbiota across different equine types in terms of bacteria, archaea and anaerobic fungi, and (ii) to determine core taxa. All of the equines studied contained archaea and anaerobic fungi as well as bacteria in their fecal microbiota. However, equine type was associated with differences in both microbial concentrations and community composition.

With respect to microbial concentrations, the largest difference observed between the equine types was in terms of the anaerobic fungi. The six-fold higher anaerobic fungal concentrations in donkey compared to horse is consistent with the reported higher fiber digestion ability of donkeys compared to horses [17]. As anaerobic fungi can take from 8 to 32 h to complete their life cycle [30], the longer fiber mean retention time in donkeys relative to that in horses is also likely to be more favorable for anaerobic fungal growth [17]. The anaerobic fungal concentration for the horse × donkey was numerically intermediate between donkey and horse. Whether this is due to horse × donkey having an intermediate fiber mean retention time compared to that of horses and donkeys is not known.

Archaeal concentrations in donkey and horse × donkey were both approximately two-fold higher compared to those in horse. As methanogenic archaea have been shown to increase the rate and extent of cellulose degradation by anaerobic fungi [31], this may result in enhanced anaerobic fungal activity and fiber degradation in donkey and horse × donkey. The archaeal and bacterial concentrations followed the same trend with equine type suggesting that the equine hindgut ecosystem may select for a certain bacteria:archaea ratio. The microbial concentrations in zebra did not significantly differ from those in horse. This is interesting as in genetic terms horse, zebras and asses represent three distinct lineages [15], and suggests that the gut physiology of zebra may be more comparable to that of horse than to that of donkey.

In general, donkey was the most distinctive among the equine types studied here. Associated with this, several bacterial genus level taxa were identified that were positively associated with donkey or the other three equine types, respectively. However, as the diet, age and management of the animals was not controlled, these initial observations regarding equine type require further investigation with more specifically designed studies. Nevertheless, the variation encompassed in the equine cohort studied here is valuable when determining the core microbiota of equine hindgut.

As with human studies [13], the number of bacterial OTUs reported to be core in the equine hindgut differs in the literature, and is influenced by a variety of factors including the number of individuals sampled as well as the approaches used to define operational taxonomic units (OTUs) and the core. The first study to report on the core bacteria in fecal samples found 123 OTUs common to four horses [3], whereas later more in depth studies reported smaller cores of 25 OTUs with ten horses/ponies [8], six OTUs with 17 horses [10], eight OTUs with 11 horses [9], 65 OTUs with 12 horses/ponies [11] and 21 OTUs with 35 ponies [7]. In this study, eight prokaryotic core OTUs were found to be shared in fecal samples from 70 different equines. These eight core OTUs represented a total of 13.4% of the prokaryotic community per animal. This is a much larger proportion of the fecal microbiota compared to the 2.3% [10] and 6.7% [7] of total sequences previously reported. This is perhaps not surprising considering that at least half of the core OTUs in this study belonged to phyla that were not detected in the core OTUs identified in the other studies.

A core OTU belonging to the phylum *Spirochaetes* was only found in the Morrison et al. [7] study, whereas no core OTU belonging to the *Verrucomicrobia* was found in any other study to date [3, 7, 8, 10, 11]. In fact, in these studies the phylum *Verrucomicrobia* was not detected at all despite it being reported in previous studies to represent 3% [32], 4.1% [2] and 18.1% [1] of the fecal microbiota of healthy horses. This phylum has also been reported to occur throughout the equine hindgut [9]. The reason for the earlier failure to detect *Verrucomicrobia* is not entirely clear, but has been previously suggested to be caused by methodological differences in the 16S rRNA gene regions and/or sequencing platforms used [9].

Following the characterization of the first cultured representative of the *Verrucomicrobia* subdivision 5 [33], this subdivision has now been re-classified as a new phylum named *Kiritimatiellaeota* [34]. One of the eight distinct phylogenetic clades within this phylum, RFP12, is comprised mainly of sequences retrieved from the intestine of vertebrates, e.g. the rumen of cattle [34] as well as equine feces [1]. Whilst the three published isolates from the phylum do not belong to the RFP12 clade, it is interesting to note that *Kiritimatiella glycovorans* has a strictly anaerobic and fermentative type of metabolism with sugars as preferred substrate [34]. The other two isolates can utilize sulfated polysaccharides including the glycoprotein mucin [35]. Manual re-annotation of the four *Verrucomicrobia* core OTUs with the latest version of the SILVA database (version 132) [36], confirmed that they do indeed belong to the *Kiritimatiellaeota* and are placed in a class called WCHB1–41. This class is named after a sequenced bacterial clone from the methanogenic zone of a contaminated aquifer [37]. In light of the sequences of Steelman et al. [1] being assigned to the phylogenetic clade RFP12 [34], it is speculated that the four *Kiritimatiellaeota* core OTUs identified in this study also belong to this RFP12 clade.

As these four core OTUs accounted for 7.2% of the mean fecal prokaryotic community/animal in this study, there is an urgent need to obtain cultured representatives of this clade in order to determine their role in the health and nutrition of mammalian herbivores. This is of particular interest, as it has been previously reported that *Verrucomicrobia* subdivision 5 was of higher relative abundance in horses suffering from laminitis compared to healthy controls [1], and that its relative abundance was dramatically decreased by the oral administration of the antibiotic trimethoprim-sulfadiazine [4].

The core OTU belonging to the *Spirochaetes* annotated as *Treponema* 2 accounted for 3.4% of the prokaryotic community/animal in this study, and at the genus level *Treponema* 2 comprised 8.4%. The genus *Treponema* was reported to be lower in relative abundance in other studies (1.9% [2] and 2.9% [1]) but consistent with this study was present in all 18 of the animals in the study of Steelman et al. [1]. In the study of Daly et al. [32], the majority of the *Spirochaetes* cloned sequences clustered with the known species *Treponema bryantii* and *Treponema succinifaciens*. Neither of these species are considered to be pathogenic, unlike several of the other 20 species listed within the *Treponema* 2 genus in the SILVA database [36]*. Treponema bryantii* utilizes fermentable substrates, in particular soluble sugars released from cellulose by cellulolytic bacteria such as *Fibrobacter* (formerly *Bacteroides*) *succinogenes* [38]. *Treponema succinifaciens* is strictly saccharolytic, can ferment pentoses, hexoses and disaccharides, and produces relatively large amounts of succinate from glucose and CO_2_ [39].

Of the three remaining core OTUs, only one could be annotated to a genus that has cultivated representatives: *Mogibacterium*. Considering that the five species belonging to this genus are all associated with oral disease [40, 41], its role in the equine hindgut ecosystem is unclear. This genus has been previously reported to occur in equine feces in some studies [2, 5, 7], but not in others [1]. This is perhaps not surprising considering this core taxon had the lowest relative abundance at both the OTU and genus level. The other two core OTUs were annotated as an unknown genus in the *Lachnospiraceae* and the uncultured genus *Ruminococcaceae* UCG − 005. Previous studies have consistently shown that bacterial OTUs belonging to the families *Lachnospiraceae* and *Ruminococcaceae* are normal members of the bacterial core of the equine hindgut [8, 10].

Only when the OTUs were grouped at the genus level were the well-known cellulolytic bacterial genera *Fibrobacter* and *Ruminococcus* 1 (containing *R. albus* and *R. flavefaciens*) found to be part of the core. Whilst it has been previously concluded that *R. flavefaciens* is more predominant in the equine hindgut than *F. succinogenes* [19], this was not indicated in the current study. The mean relative abundance of *Fibrobacter* was three times higher than that of *Ruminococcus* 1. As no single OTU belonging to these two genera was core, niche differentiation is likely to be important for the occurrence of particular OTUs. For example, in the study of Dougal et al. [10], core OTUs belonging to the family *Fibrobacteraceae* were found in animals fed a hay diet, but not with the other diets studied.

Two other bacterial genera with cultured representatives were identified as being core: *Phascolarctobacterium* and the *[Eubacterium] coprostanoligenes* group. As *Eubacterium coprostanoligenes* is a cholesterol reducing anaerobe [42], its role in the equine hindgut remains to be elucidated. The two species belonging to the genus *Phascolarctobacterium* have both been isolated from mammalian feces and utilize succinate, suggesting that this genus might also be involved in the metabolism of succinate in the equine hindgut [43, 44]. Other core genus level groupings of OTUs identified in this study belonged to uncharacterized genera in the families *Lachnospiraceae*, *Rikenellaceae* and *Ruminococcaceae*, as well as uncharacterized families in the order *Bacteroidales*. It is clear that cultured representatives of these taxa need to be isolated before it can be speculated what their role in the equine hindgut may represent.

Despite the numerous core bacterial OTUs and genera detected in this study, no core archaeal OTU or genus was observed. However, consistent with other studies the genera *Methanocorpusculum* and *Methanobrevibacter* were found to predominate [6, 27]. Both genera consist of species that mainly utilize carbon dioxide and hydrogen for the production of methane [45, 46], suggesting that functional redundancy may result in no archaeal taxon being core. This has been previously observed in ruminants where *Methanobrevibacter* is most prevalent, whereas *Methanocorpusculum* is not normally found in the rumen [47].

Similar to the archaea, no core anaerobic fungal OTU or genus was detected in this study. Findings of this study indicated that equine type was associated with differences in anaerobic fungal community composition and concentration. *Caecomyces* was predominant in the donkey where the highest anaerobic fungal concentrations were detected, whereas uncultivated AL1 predominated in the horse and zebra where the lowest anaerobic fungal concentrations were found. Further work is needed to confirm this finding with animals that are fed and managed in the same way.

The limited prevalence of *Piromyces* in the equine cohort studied here is in contrast to the fact that it has been the main genus identified in cultivation based studies to date [22, 48–51]. *Caecomyces* [52] and *Buwchfawromyces* [53] have also been cultured from equines. A culture independent survey indicated that two uncultivated taxa, currently termed AL1 and AL3 [29], were predominant in the eight equines (representing five different species) sampled [25]. A preliminary study has also shown that AL1 can be found throughout the equine hindgut [54]. As the majority of OTUs in this study could not be classified at the clade level, it is clear that novel anaerobic fungal taxa remain to be isolated from the equine hindgut.

Fecal samples are commonly used as a marker for the equine hindgut microbiota, as they can be obtained non-invasively. In terms of bacterial community composition analysis, fecal samples are generally similar to the different anatomical sections of the hindgut at the phylum and class levels [8, 9]. Indeed, fecal bacterial communities have been reported to not be significantly different from those in the colon [9], although they represent the distal regions of the hindgut to a more limited extent [8, 9, 28]. Preliminary studies have also reported differences in archaeal and anaerobic fungal community composition along the equine hindgut [54, 55]. As such, interpretation of the findings of this study relative to the proximal hindgut should be made cautiously.

Diet is also well known to influence the equine hindgut microbiome [56], and it has been previously reported that diet can influence the core microbiota [10]. Dougal et al. [10] found that animals fed a hay based diet that was either supplemented with a starch rich supplement or an oil supplement had a smaller core microbiota compared to animals fed an unsupplemented hay diet, and that only a limited core spanned all three diets. As Dougal et al. [10] did not detect any Verrucomicrobia, it is perhaps not surprising that the core present in all three of the diets provided in their study was represented by only 6 OTUs that accounted for 2.3% of total sequences. Furthermore, one Spirochaete OTU was only core in the oil supplemented hay based diet and not in the starch supplemented hay based diet or unsupplemented hay diet. It is clear from these contrasting findings, relative to the present study, that further work is needed to determine how the core microbiota in the hindgut of healthy equines is affected by diet.

## Conclusions

Equine studies to date have primarily focused on one equine species and only the bacterial component of the hindgut. In this study, observations indicated that equine type (horse, donkey, horse × donkey and zebra) was associated with differences in both fecal microbial concentrations and community composition, with donkey generally being most distinctive. Despite this, a common bacterial core was found in all the equines studied that was larger than previously reported. This was primarily due to the detection of predominant core taxa belonging to the phyla *Kiritimatiellaeota* (formerly *Verrucomicrobia* subdivision 5) and *Spirochaetes*. Archaea and anaerobic fungi were present in all animals, although no core OTU or genus was detected for either. The lack of microbial cultures representing the core and predominant bacterial and anaerobic fungal taxa, respectively, needs to be addressed, particularly as both are likely to play a key role in the ability of equines to utilize dietary fiber. There is, therefore, an urgent need to culture and characterize representative key taxa to advance fundamental understanding of the microbial taxa that underpin the equine hindgut ecosystem.

## Methods

### Animals

Fecal samples were collected from 70 different equines that were grouped into four different equine types: horse (*Equus ferus caballus*), donkey (*Equus africanus asinus*), horse × donkey (*Equus ferus caballus* × *Equus africanus asinus*) and zebra (*Equus zebra hartmannae*, *Equus quagga burchellii*, *Equus quagga boehmi* and *Equus grevyi*). The horse × donkey animals sampled could not be classed as mules or hinnies due to lack of information about their parentage. For each equine type a representative animal size range was used, with the exception of zebras where this was not possible with the exception of variation between (sub)species. All animals sampled were 4–26 years old and had been clinically healthy in the 6 months prior to sampling, with no known history of gut-related problems. Animals for each equine type were sourced from multiple locations and belonged to either The Donkey Sanctuary, Utrecht University, private owners or zoos. The majority of the animals sampled had a predominantly pasture based diet at the time of sampling, with the exception of two horses and five of the zebras, which had no access to fresh pasture. Further animal specific details are provided in Additional file 1**:** (Table S1).

### Fecal sample collection and determination of dry matter content

For each animal one freshly voided fecal sample was collected during either September or October 2016. Parts of the feces that were visibly free of dirt, bedding etc. were collected into a clean bucket, and then a pre-weighed tube was filled (approx. 20–30 g wet weight). The filled tubes were then weighed before being placed on wet ice. Samples were kept on wet ice for a maximum of one hour before being stored at − 20 °C. Fecal samples were then freeze-dried to a constant weight. For each sample, the percentage fecal dry matter content was then calculated using the original wet weight and the final freeze-dried weight.

### DNA extraction

The freeze dried fecal material was broken up by hand, and any large fibrous particles cut into smaller pieces using a sterile scalpel. The material was then placed into a mortar and manually ground with a pestle. Total DNA was extracted from 25 mg of freeze-dried and ground fecal sample using a protocol involving a combination of bead beating, Stool Transport and Recovery (STAR) buffer (Roche Diagnostics Nederland BV, Almere, Netherlands) and the Maxwell® 16 Instrument (Promega, Leiden, Netherlands). The method was as previously published [57] except that the sample was first treated for 60 s at a speed of 6.0 m/s before adding the STAR buffer, to ensure all the sample material was finely ground. The purity of the resulting DNA extract was assessed using a NanoDrop ND-1000 spectrophotometer (NanoDrop® Technologies, Wilmington, DE, USA), and the quantity assessed using a Qubit dsDNA BR assay (Thermoscientific, Bleiswijk, Netherlands).

### Determination of microbial concentrations

For absolute quantification of bacteria and archaea, SYBR green qPCR assays were performed with sample DNA extracts using a CFX384 Touch™ Real-Time PCR Detection System (Bio-Rad Laboratories BV, Veenendaal, Netherlands) as previously described [57]. All qPCR analyses were carried out in triplicate with a reaction volume of 10 μL. Bacterial and archaeal assays used 0.2 ng and 2 ng, respectively, of sample DNA. Equine specific standard curves (10^8^ to 10^2^ amplicon copies/μL) for the assays were prepared using purified PCR amplicons generated from an equine fecal DNA extract using the primers and cycling conditions previously described for the preparation of standards [57].

For absolute quantification of anaerobic fungi, a Taqman probe based method was used as previously described [58] with the exception that a CFX384 Touch™ Real-Time PCR Detection System (Bio-Rad Laboratories BV) was used. All qPCR analyses were carried out in triplicate with a reaction volume of 10 μL, and 2 ng of sample DNA was used. Standard curves (10^8^ to 10^1^ amplicon copies/μL) for the assays were prepared using purified PCR amplicons generated from *Neocallimastix frontalis* strain R_E_1 DNA (kindly provided by Dr. Tony Callaghan, Bavarian State Research Center for Agriculture, Freising, Germany). The PCR amplicon was generated using the primers Neo18SF (5′-AATCCTTCGGATTGGCT-3′: [58]) and AF LSU reverse (5′-CTTGTTAAMYRAAAAGTGCATT-3′: [59]).

### Prokaryotic community composition analysis

For 16S rRNA gene based prokaryotic composition profiling, barcoded amplicons from the V4 region of 16S rRNA genes were generated from the DNA extracts using a 2-step PCR strategy as previously described [57]. Barcoded PCR products were mixed in equimolar amounts into pools together with defined synthetic mock communities which allow assessment of potential technical biases [60]. Pools were then sequenced on the Illumina HiSeq platform using the HiSeq Rapid Run 300 bp paired end (PE) sequencing mode (GATC-Biotech, Konstanz, Germany, now part of Eurofins Genomics Germany GmbH).

The 16S rRNA gene sequencing data was analyzed using NG-Tax [60]. NG-Tax defines OTUs using an open reference approach, and OTUs are defined as unique sequences that are above a user-defined minimum abundance threshold. NG-Tax (version NG-Tax-1.jar, which is available at http://download.systemsbiology.nl/ngtax/) was run with the following default settings: 70 nt read length, ratio OTU abundance 2.0, classify ratio 0.8, minimum percentage threshold 0.1%, identity level 100% and error correction of 1 mismatch (98.5%). Paired-end libraries were filtered to contain only read pairs with perfectly matching barcodes, and those barcodes were used to demultiplex reads by sample. Taxonomy was assigned to OTUs using the 128 version of the SILVA 16S rRNA gene reference database [36].

### Anaerobic fungal composition analysis

For anaerobic fungal community composition profiling, barcoded amplicons comprising the partial 18S rRNA gene (~ 130 bp), full ITS1 region and partial 5.8S rRNA gene (~ 31 bp) were generated using a 2-step PCR strategy with a SensoQuest Labcycler (Göttingen, Germany) [61]. The first PCR step was performed with previously published ARISA primers [58] with the addition of UniTag adapters (underlined): Neo 18S For 5′-GAGCCGTAGCCAGTCTGCAATCCTTCGGATTGGCT-3′ and Neo 5.8S Rev. 5′-GCCGTGACCGTGACATCGCGAGAACCAAGAGATCCA-3′. PCR was performed in a total volume of 25 μL containing 1× HF buffer (Finnzymes, Vantaa, Finland), 1 μL dNTP Mix (10 mM; Promega), 1 U of Phusion® Hot Start II High-Fidelity DNA polymerase (Finnzymes), 500 nM of each primer and 2 ng of sample DNA. The cycling conditions consisted of an initial denaturation at 98 °C for 3 min followed by 40 cycles of 98 °C for 10 s, 58 °C for 30 s and 72 °C for 30 s, and then a final extension at 72 °C for 6 min. Triplicate PCR reactions were prepared for each sample, along with a non-template control (NTC) reaction. The presence of the PCR products was assessed by agarose gel electrophoresis on a 2% (w/v) agarose gel containing 1× SYBR® Safe (Invitrogen, Carlsbad, CA, USA). Pooled triplicate reactions, as well as the negative NTC reaction, were then purified using HighPrep™ (MagBio Europe Ltd., Kent, United Kingdom).

The second PCR step was then employed to add an 8 nucleotide sample specific barcode to the 5′- and 3′- end of the PCR products as previously described [57]. Each PCR reaction, with a final volume of 100 μL, contained 5 μL of the purified first step PCR product, 5 μL each of barcoded forward and reverse primers (10 μM), 2 μL dNTP Mix (10 mM), 2 U of Phusion® Hot Start II High-Fidelity DNA polymerase and 1× HF buffer. Amplification consisted of an initial denaturation at 98 °C for 30 s followed by 5 cycles of 98 °C for 10 s, 52 °C for 20 s and 72 °C for 20 s, and then a final extension at 72 °C for 10 min. Barcoded PCR products were then purified using the HighPrep™ and quantified using a Qubit in combination with the dsDNA BR Assay Kit (Invitrogen). Purified barcoded PCR products were then pooled in equimolar amounts along with defined synthetic mock communities [61]. Pools were then sequenced on the Illumina HiSeq platform using the HiSeq Rapid Run 300 bp PE sequencing mode (GATC-Biotech, Konstanz, Germany, now part of Eurofins Genomics Germany GmbH).

The anaerobic fungal sequence data was then analyzed using NG-Tax [61]. NG-Tax (version NGTax-2.jar which is available at http://download.systemsbiology.nl/ngtax/) was run using the default parameters (as described earlier) except for the following: 150 nt read length, minimum percentage threshold 0.6% and error correction of 1 mismatch (99.3%). As the barcoded amplicon primers used were not within the AF-ITS1 database used for OTU annotation (which is a requirement for annotation by NG-Tax), an empty database file (emptydb.fasta.gz which is available at http://download.systemsbiology.nl/ngtax/databases/) was used and the OTUs then subsequently annotated manually.

Fasta files of the OTUs from the NG-Tax generated biom file were extracted using the script OTUseq_export.py. The OTUs were annotated using BLASTN searches against the AF-ITS1 database [29] (version 3.3, available from www.anaerobicfungi.org) using default settings with “-num_alignments 10” (BLAST version 2.4.0). For OTUs that could not be annotated by the AF-ITS1 database, BLASTN searches were performed against the NCBI database. Cut-off levels for OTU annotations were determined based on the mean percentage similarities of full-length sequences in the AF-ITS1 database within clade and within genus. These cut-off levels were > 98% for clade and > 95% for genus. The NG-Tax generated biom file was converted to a tab delimited table to enable OTU annotations to be added. The OTUs that were clearly associated with the NTC sample were also manually removed from the tab delimited table at this stage, along with any OTUs that were not anaerobic fungal in origin. The resulting tab delimited table was then converted back to a biom file.

### Statistical analysis

Microbial composition summary box plots, alpha diversity, UniFrac based Principal Coordinate Analysis (PCoA) and core microbiome analysis was performed within R (version 3.4.1) [62] using the following packages: microbiome (http://microbiome.github.com/microbiome), microbiomeutilities (https://github.com/microsud/microbiomeutilities), RColorBrewer [63], bindrcpp (https://github.com/krlmlr/bindrcpp), magrittr [64], phyloseq [65], picante [66], nlme [67], vegan [68], lattice [69], permute [70], ape [71], ggplot2 [72] and ggpubr [73]. The figures from ggplot2 and ggpubr were further refined in Adobe Illustrator CC (version 22.1) to improve their clarity. Redundancy analysis (RDA) was performed using Canoco 5 [74] to assess the relationship between genus-level phylogenetic groupings of the OTUs and equine type. Univariate data (fecal dry matter, alpha diversity and qPCR data) were analysed using a one-way ANOVA with equine type as a single independent factor and using a Tukey post-hoc test (Genstat 18th Edition, VSN International Ltd). Fecal dry matter and alpha diversity data was normally distributed, whereas the qPCR data was not normally distributed and, therefore, was analysed after Log_10_ transformation.

## Supplementary information

**Additional file 1: Table S1.** Details of the animals used in the study.

**Additional file 2: Figure S1.** Effect of equine type on fecal dry matter content. Columns represent the mean (*n* = 18, except for zebra where *n* = 16) and error bars the SEM. Letters indicate significant differences (*P* < 0.05).

**Additional file 3: Figure S2.** Effect of equine type on the fecal bacterial (A), anaerobic fungal (B) and archaeal (C) concentrations on a fresh weight basis. Columns represent the mean (n = 18, except for zebra where n = 16) and error bars the SEM. Letters above the bars within each plot indicate significant differences (*P* < 0.05). Percentages stated on the x-axis in brackets indicate how the mean of each equine type compares to that of the horse.

**Additional file 4: Figure S3.** Boxplot showing the six main bacterial and archaeal phyla detected in the different equine types (minor phyla grouped as ‘Other’).

**Additional file 5: Figure S4.** Boxplot showing the twenty bacterial and archaeal families detected in the different equine types that were of highest relative abundance. Families are grouped by phylum as follows: (1) *Bacteroidetes*, (2) *Euryarchaeota*, (3) *Fibrobacteres*, (4) *Firmicutes*, (5) *Spirochaetae* and (6) *Verrucomicrobia*. Families that could not be classified are grouped as ‘Unclassified Families’.

**Additional file 6: Figure S5.** Boxplot showing the anaerobic fungal clades detected in the different equine types. Clades that could not be classified are grouped as ‘Unclassified Clades’.

**Additional file 7: Figure S6.** Boxplot showing the anaerobic fungal genera detected in the different equine types. Genera that could not be classified are grouped as ‘Unclassified Genera’.

## Data Availability

The datasets and material supporting the conclusions of this article are provided as follows. Additional information is provided in Additional files 1, 2, 3, 4, 5, 6 and 7. The barcoded amplicon sequence data is deposited in the European Nucleotide Archive under the study accession number PRJEB31377. All the sample barcodes, R codes, data used in the analysis and the workflow (as Rmarkdown files to reproduce the microbial community analysis) are available at https://github.com/mibwurrepo/EdwardsJ_2019_EquineCoreMicrobiome.

## Abbreviations

AF-ITS1: Anaerobic fungal ITS1
ITS1: Internal Transcribed Spacer 1
NTC: Non-template control
OTU: Operational Taxonomic Unit
PCoA: Principal co-ordinate analysis
PCR: Polymerase chain reaction
PE: Paired end
qPCR: Quantitative PCR
RDA: Redundancy analysis
SD: Standard deviation
SEM: Standard error of the mean
