# Supplementary material for: Multi-kingdom characterization of the core equine fecal microbiota based on multiple equine (sub)species
Source: Anim Microbiome. 2020 Feb 12;2:6. doi: 10.1186/s42523-020-0023-1 (PMC7807809; doi:10.1186/s42523-020-0023-1)
Supplement: Supplementary file 2 — Additional file 2: Figure S1. Effect of equine type on fecal dry matter content. Columns represent the mean (n = 18, except for zebra where n = 16) and error bars the SEM. Letters indicate significant differences (P < 0.05). [file 42523_2020_23_MOESM2_ESM.pdf]

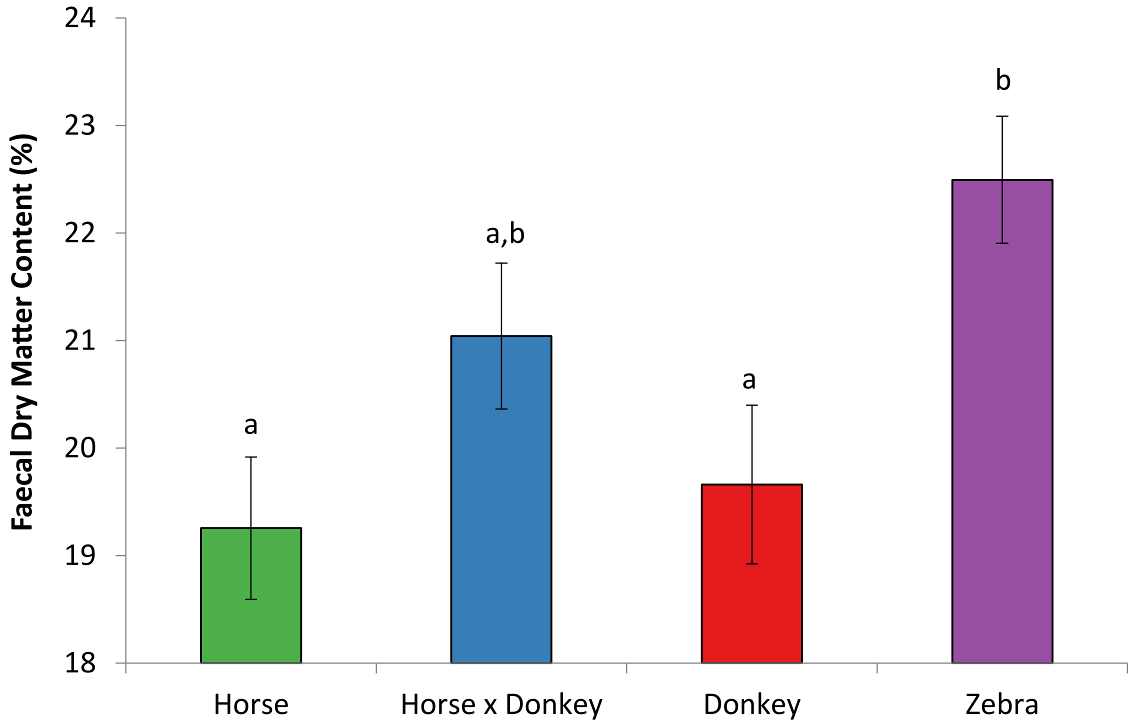

Figure S1. Effect of equine type on faecal dry matter content. Columns represent the mean (n=18, except for zebra where n=16) and error bars the SEM. Letters indicate significant differences ($P < 0.05$).
