# Supplementary material for: Multi-kingdom characterization of the core equine fecal microbiota based on multiple equine (sub)species
Source: Anim Microbiome. 2020 Feb 12;2:6. doi: 10.1186/s42523-020-0023-1 (PMC7807809; doi:10.1186/s42523-020-0023-1)
Supplement: Supplementary file 3 — Additional file 3: Figure S2. Effect of equine type on the fecal bacterial (A), anaerobic fungal (B) and archaeal (C) concentrations on a fresh weight basis. Columns represent the mean (n = 18, except for zebra where n = 16) and error bars the SEM. Letters above the bars within each plot indicate significant differences (P < 0.05). Percentages stated on the x-axis in brackets indicate how the mean of each equine type compares to that of the horse. [file 42523_2020_23_MOESM3_ESM.pdf]

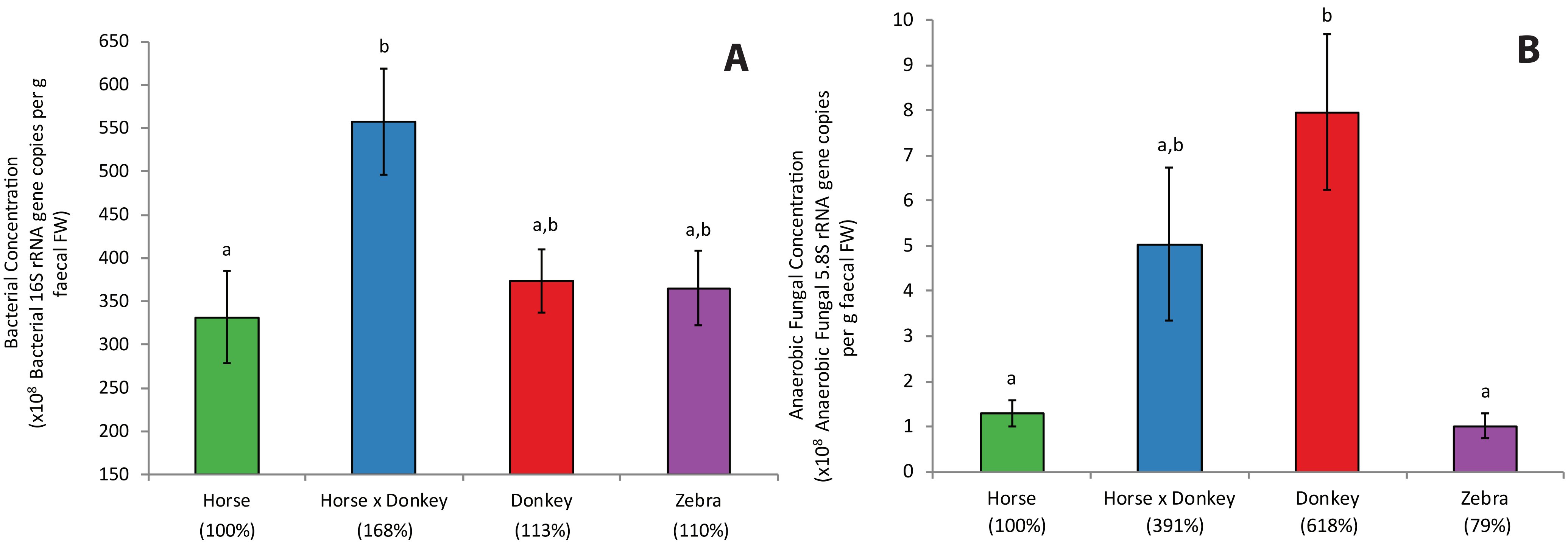

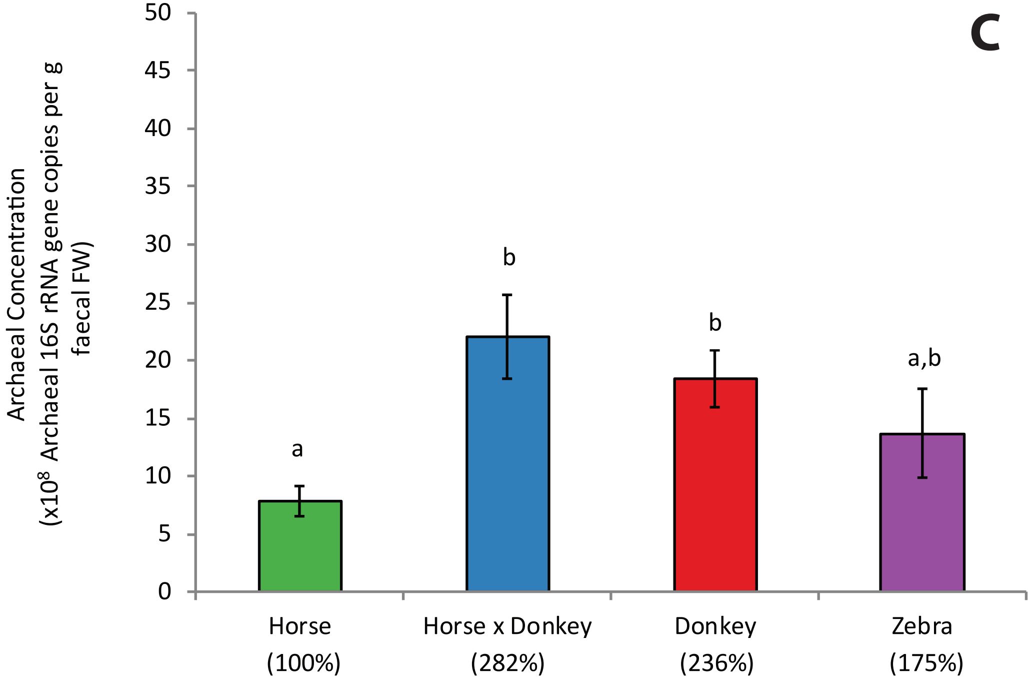

Additional File 3:Figure S2. Effect of equine type on the faecal bacterial (A), anaerobic fungal (B) and archaeal (C) concentrations on a fresh weight basis. Columns represent the mean (n=18, except for zebra where n=16) and error bars the SEM. Letters above the bars within each plot indicate significant differences ($P < 0.05$). Percentages stated on the x-axis in brackets indicate how the average of each equine type compares to that of the horse.
