# Supplementary material for: Multi-kingdom characterization of the core equine fecal microbiota based on multiple equine (sub)species
Source: Anim Microbiome. 2020 Feb 12;2:6. doi: 10.1186/s42523-020-0023-1 (PMC7807809; doi:10.1186/s42523-020-0023-1)
Supplement: Supplementary file 4 — Additional file 4: Figure S3. Boxplot showing the six main bacterial and archaeal phyla detected in the different equine types (minor phyla grouped as ‘Other’). [file 42523_2020_23_MOESM4_ESM.pdf]

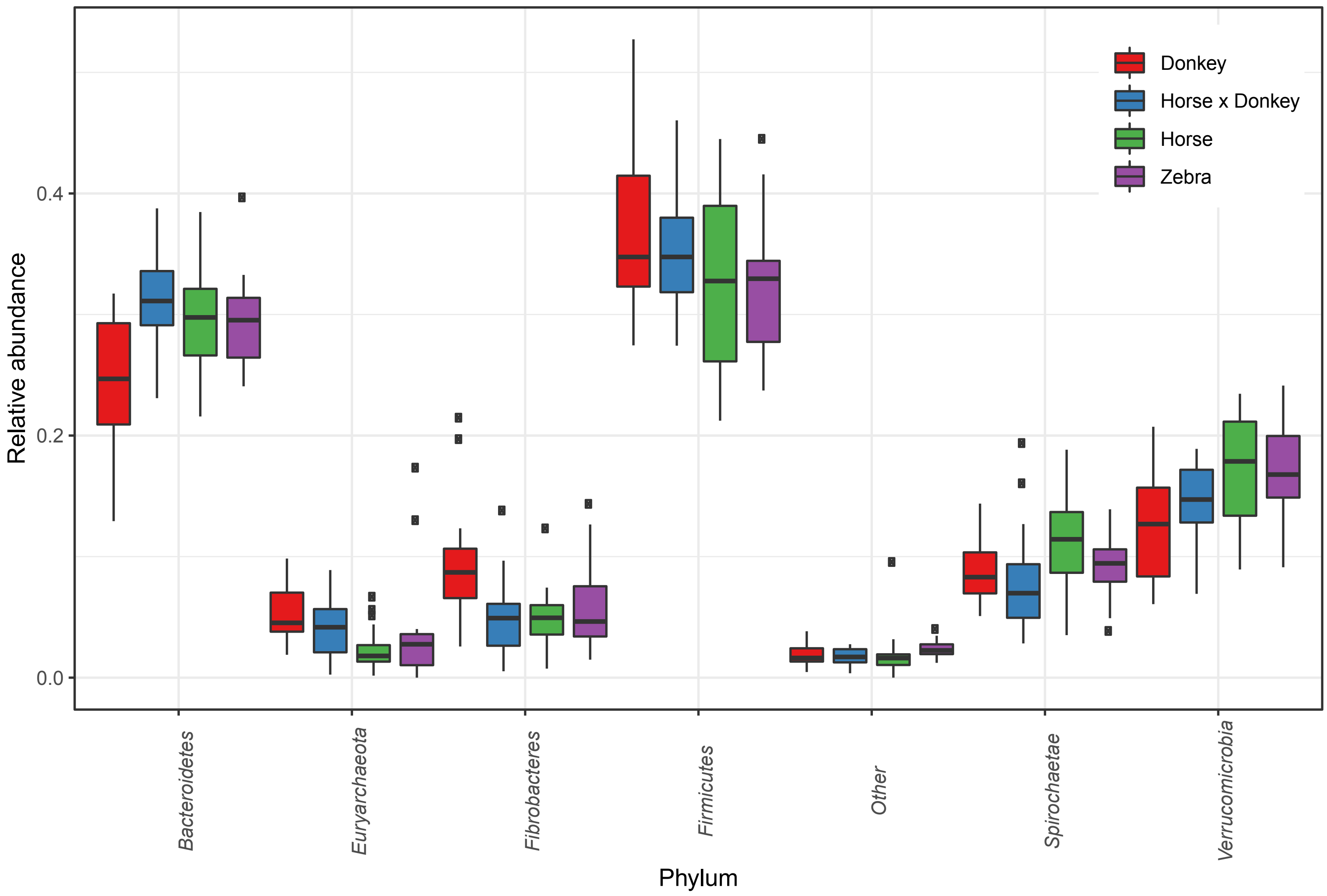

Figure S3: Boxplot showing the six main bacterial and archaeal phyla detected in the different equine types (minor phyla grouped as 'Other').
