# Supplementary material for: Multi-kingdom characterization of the core equine fecal microbiota based on multiple equine (sub)species
Source: Anim Microbiome. 2020 Feb 12;2:6. doi: 10.1186/s42523-020-0023-1 (PMC7807809; doi:10.1186/s42523-020-0023-1)
Supplement: Supplementary file 5 — Additional file 5: Figure S4. Boxplot showing the twenty bacterial and archaeal families detected in the different equine types that were of highest relative abundance. Families are grouped by phylum as follows: (1) Bacteroidetes, (2) Euryarchaeota, (3) Fibrobacteres, (4) Firmicutes, (5) Spirochaetae and (6) Verrucomicrobia. Families that could not be classified are grouped as ‘Unclassified Families’. [file 42523_2020_23_MOESM5_ESM.pdf]

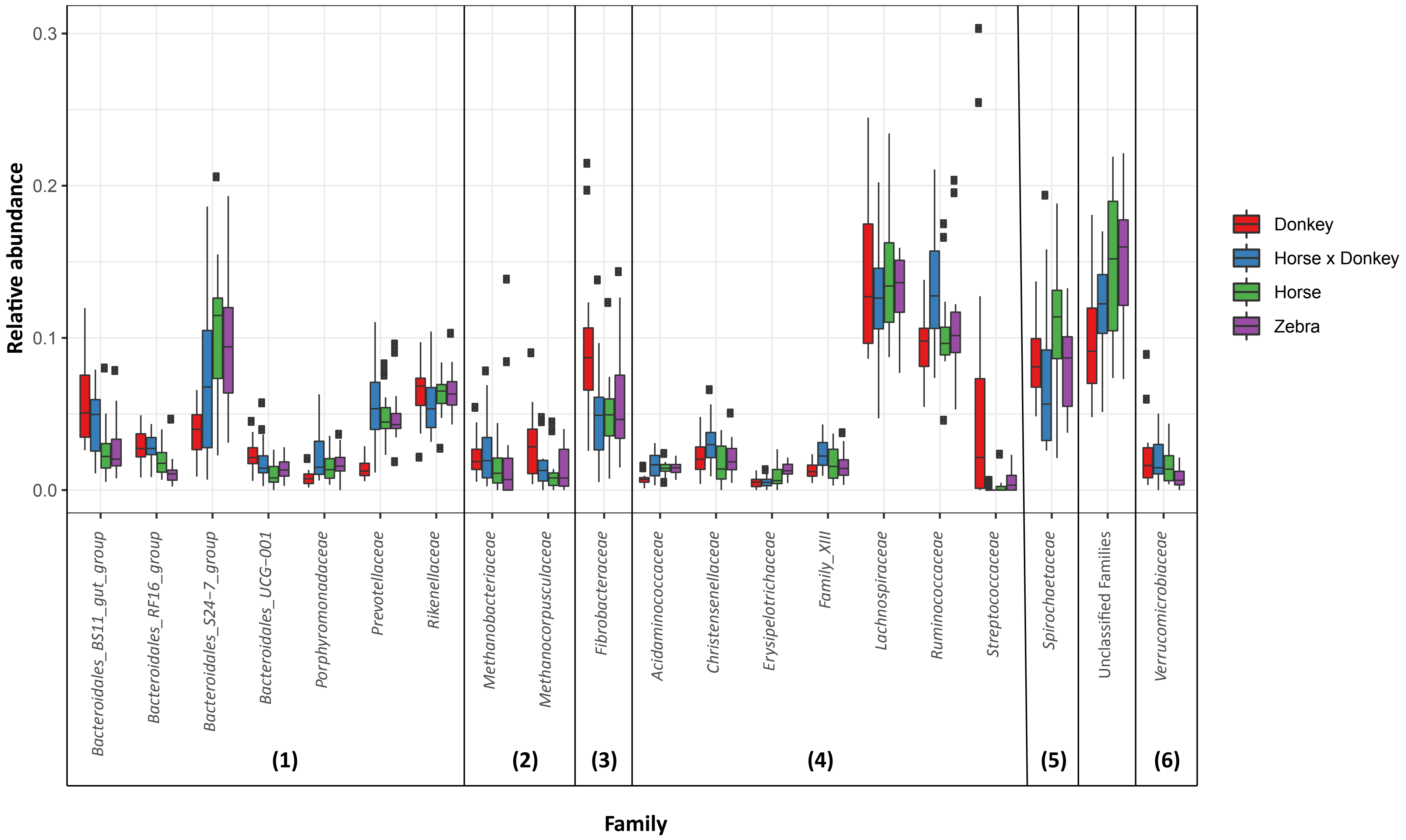

Additional File 5:Figure S4. Boxplot showing the twenty bacterial and archaeal families detected in the different equine types that were of highest relative abundance. Families are grouped by phylum as follows: (1) *Bacteroidetes*, (2) *Euryarchaeota*, (3) *Fibrobacteres*, (4) *Firmicutes*, (5) *Spirochaetae* and (6) *Verrucomicrobia*. Families that could not be classified are grouped as ‘Unclassified Families’.
