# Supplementary material for: Multi-kingdom characterization of the core equine fecal microbiota based on multiple equine (sub)species
Source: Anim Microbiome. 2020 Feb 12;2:6. doi: 10.1186/s42523-020-0023-1 (PMC7807809; doi:10.1186/s42523-020-0023-1)
Supplement: Supplementary file 6 — Additional file 6: Figure S5. Boxplot showing the anaerobic fungal clades detected in the different equine types. Clades that could not be classified are grouped as ‘Unclassified Clades’. [file 42523_2020_23_MOESM6_ESM.pdf]

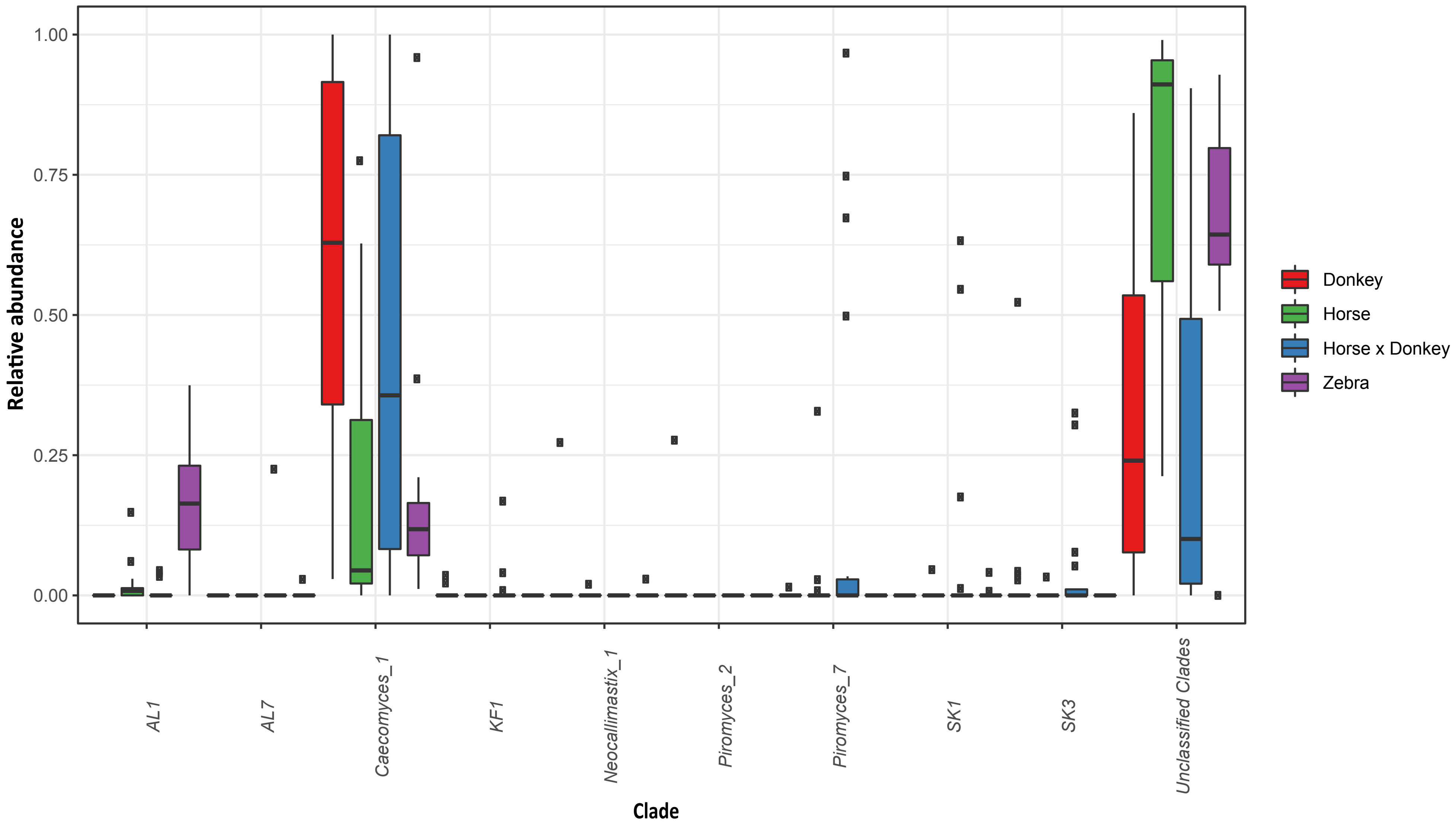

Figure S5: Boxplot showing the anaerobic fungal clades detected in the different equine types. Clades that could not be classified are grouped as 'Unclassified Clades'.
