# Supplementary material for: Multi-kingdom characterization of the core equine fecal microbiota based on multiple equine (sub)species
Source: Anim Microbiome. 2020 Feb 12;2:6. doi: 10.1186/s42523-020-0023-1 (PMC7807809; doi:10.1186/s42523-020-0023-1)
Supplement: Supplementary file 7 — Additional file 7: Figure S6. Boxplot showing the anaerobic fungal genera detected in the different equine types. Genera that could not be classified are grouped as ‘Unclassified Genera’. [file 42523_2020_23_MOESM7_ESM.pdf]

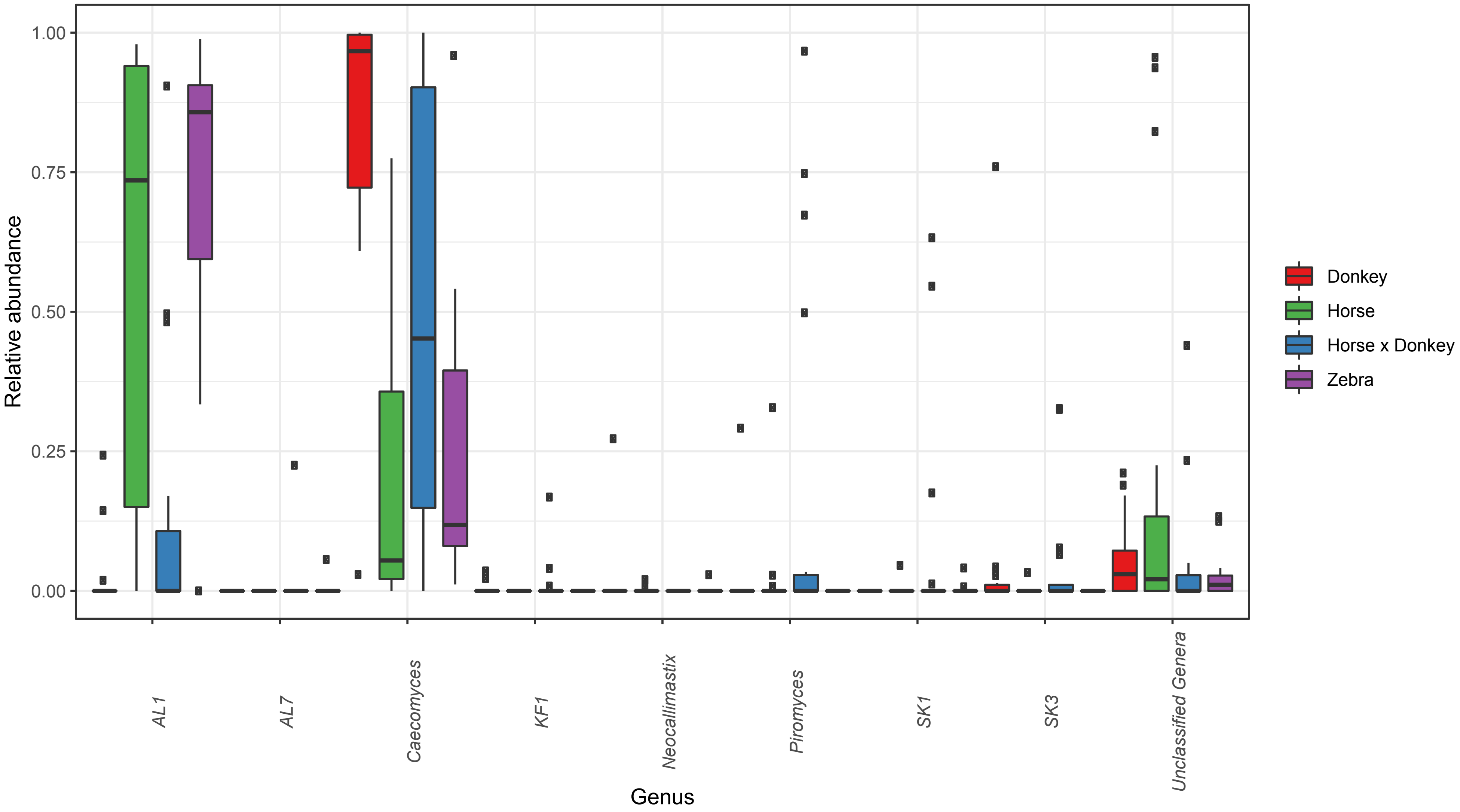

Figure S6: Boxplot showing the anaerobic fungal genera detected in the different equine types. Genera that could not be classified are grouped as 'Unclassified Genera'.
